# The Impact of Genetic Polymorphisms on Genotoxicity (DNA Damage) Among Children Exposed to Environmental Mutagens: A Systematic Review

**DOI:** 10.1002/jat.70216

**Published:** 2026-04-28

**Authors:** Thiago Guedes Pinto, Daniel Vitor de Souza, Gabriel Carvalhal de Aguiar, Ana Claudia Muniz Renno, Patricia Ramos Cury, Jean Nunes dos Santos, Rogerio Aparecido Dedivitis, Daniel Araki Ribeiro

**Affiliations:** ^1^ Department of Biosciences, Institute of Health and Society Federal University of São Paulo, UNIFESP Santos São Paulo Brazil; ^2^ Department of Periodontics, School of Dentistry Federal University of Bahia, UFBA Salvador Bahia Brazil; ^3^ Department of Head and Neck Surgery University of Sao Paulo, USP São Paulo Brazil

**Keywords:** children, DNA damage, environmental mutagens, genetic polymorphism, genotoxicity

## Abstract

Children, as a particularly vulnerable group, are more susceptible to these mutagens due to their developing immune systems, higher physiological vulnerability, and increased exposure through behaviors like outdoor play and hand‐to‐mouth activities. This review aims to investigate the relationship between these environmental exposures, genetic polymorphisms, and DNA damage in children, with a focus on how genetic factors influence the extent of damage caused by these mutagens. After literature review, a total of 10 studies were thoroughly reviewed by four independent reviewers, who assessed each study according to specific evaluation criteria. Our findings indicated a significant association between genetic polymorphisms and genotoxic effects in individuals exposed to environmental mutagens, with nine studies (out of 10) reporting positive correlations between genotoxicity and polymorphisms in genes involved in DNA repair and metabolism. In summary, individuals with specific genotypes may exhibit varying levels of DNA damage. Regarding quality assessment, nine of the studies (9 out of 10) were classified as strong or moderate, indicating a significant need for improved methodological rigor in future research. Collectively, these findings support the idea that the genotoxic effects of various environmental mutagens in children are heavily influenced by genetic polymorphisms.

## Abstract 2

Children and adolescents are highly vulnerable to environmental mutagens due to developmental and behavioral factors. This review examined associations between exposure to mutagens (e.g., PAHs, benzene, radiation, and pesticides), genetic polymorphisms, and DNA damage. Ten studies were analyzed, with most showing positive correlations between polymorphisms in DNA repair and metabolism genes and genotoxic effects. Findings suggest that genetic variability influences susceptibility to DNA damage, highlighting the role of genotype in modulating mutagen‐related risks in young populations.

## 1 Introduction

The rapid industrialization and urbanization of the modern world have led to an alarming escalation in the levels of environmental pollutants, including polycyclic aromatic hydrocarbons (PAHs), benzene, radiation, and pesticides, all of which pose significant risks to public health, particularly in vulnerable populations, such as children (Smith et al. 2020). These environmental contaminants are widespread, found in air, soil, and water near industrial complexes, agricultural fields, and urban areas, contributing to the growing burden of genotoxic exposures (Smith et al. 2020).

Among the most concerning pollutants are PAHs and benzene, which are known to have genotoxic and carcinogenic properties (López et al. 2019). PAHs are primarily produced during the incomplete combustion of organic materials and are prevalent in urban areas with heavy industrial activity and traffic (Miller et al. 2017). Similarly, benzene, a volatile organic compound, is widely found in vehicle exhaust, industrial emissions, and tobacco smoke, all of which contribute to its environmental presence (Garcia et al. 2020). Radiation from both natural and man‐made sources further exacerbates the risk, especially in areas with nuclear power plants, mining activities, or industrial waste sites (Gonzalez et al. 2021). Pesticides, often used in agriculture, are also a significant source of environmental contamination, contributing to the spread of toxic chemicals into the air, water, and soil, which then enter the food chain (Martin et al. 2019).

The physiological effects of these exposures are largely mediated by the metabolism of these compounds in the human body, a process influenced by enzymes such as cytochrome P450s (CYP) and glutathione‐S‐transferases (GST), which play essential roles in detoxifying harmful substances (Anderson et al. 2016). Genetic polymorphisms in the genes encoding these enzymes can alter their functionality, thereby influencing the activation and deactivation of toxic compounds, such as PAHs and pesticides, and modulating the degree of genetic damage (Taylor et al. 2018). These genetic variations contribute to inter‐individual differences in susceptibility to DNA damage induced by environmental pollutants (Thompson et al. 2019).

Children are particularly susceptible to these toxicants due to their higher physiological vulnerability, developmental processes, and behavioral patterns, such as increased outdoor activity and hand‐to‐mouth behavior, which increases their exposure to environmental pollutants (Jones et al. 2018). Some authors have demonstrated that children residing in areas with high exposure to these pollutants often show higher levels of some biomarkers for DNA damage, such as 8‐hydroxy‐2‐deoxyguanosine (8‐OHdG) and comet assay parameters, such as tail moment (Williams et al. 2020). These assays indicate the presence of oxidative stress and genetic alterations that may lead to long‐term health effects, including cancer (Chavez et al. 2021). However, the role of genetic polymorphisms in key metabolic enzymes, such as CYP1A1, CYP1B1, and GST, in modulating these effects remains insufficiently explored, especially in children (Sanchez et al. 2022).

As a result and because of the insufficient scientific evidence, this study aims to further explore the relationship between environmental exposures, genetic polymorphisms, and DNA damage in children exposed to various environmental mutagens. By understanding how genetic factors influence the metabolism of environmental pollutants and contribute to the extent of genetic damage, we hope to provide a clearer picture of the risks to children. Additionally, this systematic review seeks to assess the quality of the studies included to confirm the reliability of the results. As a result, it explores the influence of genetic variations on the development of genotoxicity in populations exposed to these mutagens. Such investigations may provide valuable insights into factors that contribute to individual susceptibility to genetic damage in developing individuals, which is crucial for human biomonitoring initiatives in children.

## 2 Material and Methods

### 2.1 Eligibility Criteria

This systematic review was conducted in accordance with the criteria established in the 2020 Preferred Reporting Items for Systematic Reviews and Meta‐Analyses (PRISMA) guidelines. In addition, the PICOS strategy was employed to organize this review. Using this approach, we defined the elements of the PICOS framework as follows: Population (P) refers to children (oral mucosa or peripheral blood cells); Intervention (I) encompasses environmental mutagens such as radiation, pollution, and pesticides; Comparison (C) involves control groups (children as well); Outcome (O) focuses on genotoxicity related to genetic polymorphisms; and Study design (S) includes biomonitoring studies.

Studies were deemed eligible for inclusion in our analysis if they satisfied the following criteria: (1) in vivo evaluations of genetic damage in children; (2) research involving human subjects (children) exposed to any form of environmental mutagen; (3) publications written in English; (4) inclusion of genetic polymorphism analysis; and (5) provision of data consistent with established scientific standards.

Conversely, studies were excluded if they met any of the following criteria: (1) they were conference abstracts, reviews, editorials, or letters; (2) the full text was unavailable in English; (3) data were inaccessible or could not be extracted, or if the study involved combined exposures with other substances; (4) they were multigenerational studies; (5) they did not include a genotoxicity assessment; (6) they did not address genetic polymorphisms in children exposed to environmental mutagens (such as radiation, pollutants, or pesticides); or (7) they presented incomplete or ambiguous results.

### 2.2 Data Search

In March 2025, we conducted a thorough search across PubMed, SCOPUS, and Web of Science to identify relevant articles using the following search terms and Boolean operators: (“Environmental mutagens”) AND (“Genotoxicity” OR “DNA damage” OR “genetic damage” OR “DNA breakage” OR “genetic injury” OR “DNA injury” OR “chromosome damage”) AND (“Micronucleus assay”) AND (“genetic polymorphism”) AND (“children”). Additionally, we manually reviewed references and related citations. The effectiveness of our search terms was validated by ensuring they retrieved a comprehensive range of relevant literature.

Searches were restricted to English‐language publications, with no date limitations. Abstracts were individually reviewed by four reviewers (T.G.P., D.V.S., G.C.A., and D.A.R.), and full‐text assessments were performed to confirm eligibility. Discrepancies among reviewers were resolved through discussion, ultimately reaching a consensus. The detailed data strategy is presented in Table 1.

**TABLE 1:** Electronic databases used and search strategy.

Electronic databases used	Search strategy (March, 2025)
PubMed https://www.ncbi.nlm.nih.gov/pubmed/Scopus https://www.scopus.com Web of Science https://www.webofscience.com/wos/alldb/basic‐search	(Environmental pollutants) OR (Radiation) OR (Pesticide) OR (polycyclic aromatic hydrocarbons) OR (PAHs) OR (Pollution) OR (benzene), AND (DNA Damages) OR (Damage, DNA) OR (Damages, DNA) OR (DNA Injury) OR (DNA Injuries) OR (Injuries, DNA) OR (Injury, DNA) OR (Genotoxic Stress) OR (Genotoxic Stresses) OR (Stresses, Genotoxic) OR (Genotoxicity) OR (Mutagenicity) OR (comet assay) OR (micronucleus assay) OR (Stresses, Genotoxic) OR (micronucleus) OR (micronucleated cell) OR (chromosome damage) OR (chromosomal injury) OR (chromosome breakage) OR (chromosome aberration test) OR (sister chromatid exchange test) OR (8‐ohdg) AND (genetic polymorphism) OR (gene polymorphism) OR (polymorphism) OR (polymorphic gene) OR (gene polymorphic) OR (gene, polymorphism) OR (gene, polymorphic) OR (Genetic variation) OR (Gene variation) AND (children) OR (Child).

### 2.3 Data Extraction and Quality Assessment

Four reviewers (T.G.P., D.V.S., G.C.A., and D.A.R.) independently evaluated studies for eligibility by reviewing titles, abstracts, and full texts. Any disagreements among reviewers were resolved through a consensus‐driven approach. The data extracted from the selected studies encompassed the following details: authors, publication year and country, cell types investigated, children's age, duration of exposure, type of assay used, the number of cells analyzed, genotoxicity assessment method, blinding techniques, statistical methods employed, inclusion of negative controls, doses administered, environmental mutagen involved, and key findings.

Similarly, the quality of the included studies was independently assessed by the same four reviewers (T.G.P., D.V.S., G.C.A., and D.A.R.). During this evaluation, all potential confounding variables were carefully considered. A study was classified as STRONG in its final evaluation if it accounted for all identified variables. If fewer than one variable was controlled for, the study was rated as MODERATE. In contrast, studies that failed to address two or more variables were categorized as WEAK, following the criteria established by Guedes Pinto et al. (2024).

## 3 Results

### 3.1 Study Selection

The initial online search yielded a total of 301 scientific articles. From this pool, 285 duplicates were identified and removed. Following a review of titles and abstracts, six articles were found to be unrelated to the study's objectives and were excluded. These excluded studies encompassed reviews, case reports, commentaries, editorials, non‐English language papers, and letters to the editor. The full texts of the remaining 10 studies were thoroughly analyzed by the authors of this review. The study selection process is depicted in the flow chart shown in Figure 1, as presented by the authors of this paper.

**FIGURE 1:**
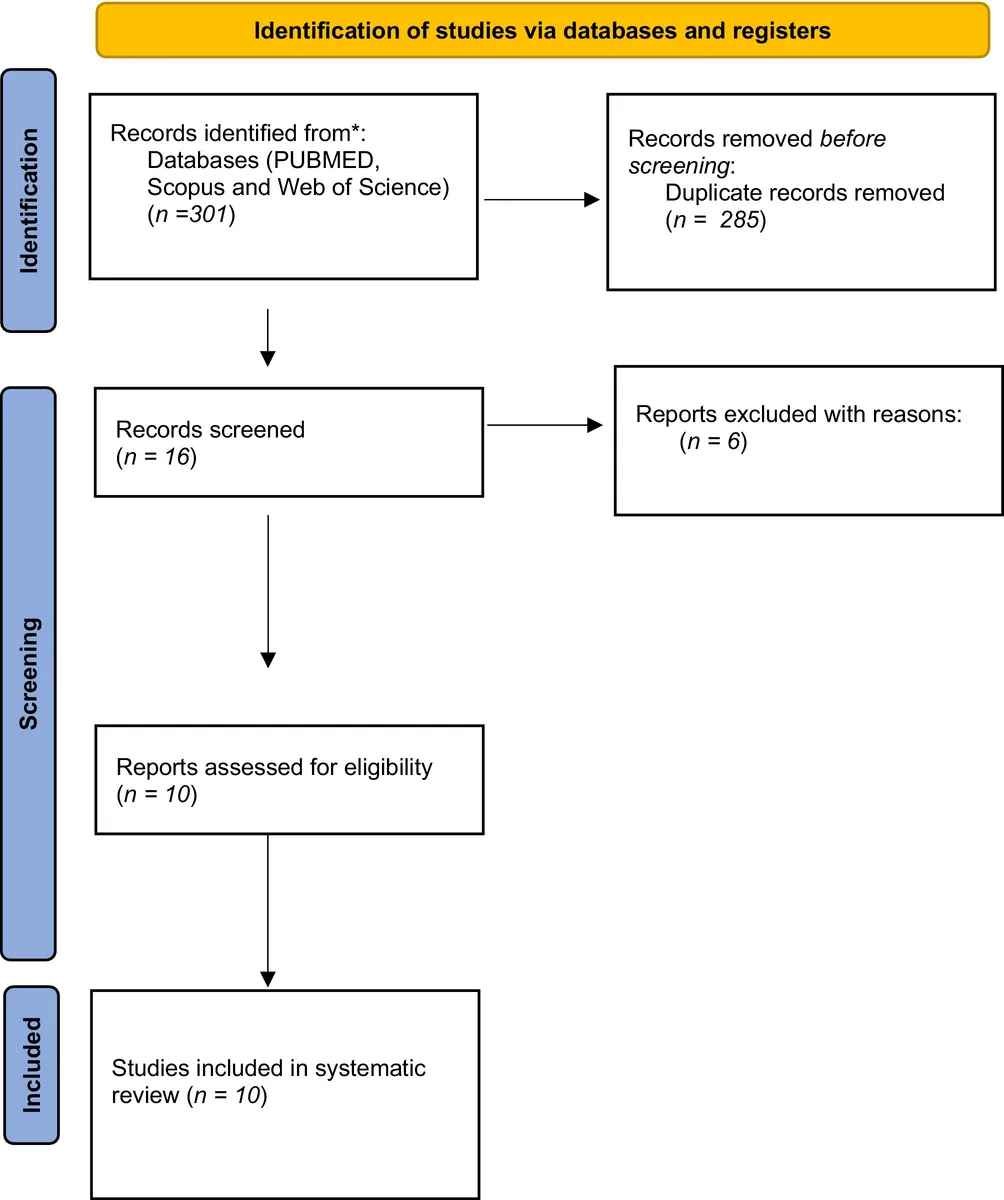
The flow chart of the study investigating the role of genetic polymorphisms in children exposed to environmental mutagens.

Table 2 provides a thorough summary of the essential characteristics of the studies evaluated. A total of 10 studies were reviewed, with three conducted in Mexico and one each in Colombia, China, Taiwan, Russia, Poland, Germany, and Thailand. The publication years of the selected articles range from 2008 to 2024. All of the studies centered on mutagen exposure in children. For more detailed information, please refer to Table 2.

**TABLE 2:** Key characteristics of the studies included in this systematic review.

Author	Year of publication	Country	Age	*N*/gender/group	Exposure	Gene polymorphism investigated
Marín et al.	2024	Colombia	Median age was 3.58 years	*n* = 416,175 in Bogota and 241 in Medellín (53% of the children were females, and 47% were males)	Airborne particulate matter (PM2.5) and Nitrogen dioxide—air pollution (NO_2_); (also considered: in‐house cockroaches; lifestyle like cereal and artificially sweetened soft drinks consumption in the diet)	*GSTT1* *GSTM1* *CYP1A1* *H2AX* *OGG1* *SOD2*
Sun et al.	2022	China	5–6 years old	*n* = 86 Control group (Campus B) *n* = 40 13 females and 27 males Exposed group (Campus A) *n* = 46 23 females and 23 males	Airborne particulate matter (PM2.5) interaction with gut microbiota	*OXTR*
Chang et al.	2021	Taiwan	4–15 years	*n* = 181 85 children with ADHD 96 control group	Organophosphate pesticides and attention‐deficit/hyperactivity disorder children	*PON*
Montero‐Montoya et al.	2020	Mexico	Control group – Ixtenco City 9.6 ± 1.4 (7–12 years) Exposed Group—Tepetitla City 10.5 ± 1.4 (7–13 years)	*N* = 184 Control group—Ixtenco City (93%–52% males and 48% females) Exposed group—Tepetitla City (91%–48% males and 52% females)	Several = polycyclic aromatic hydrocarbons, benzene, toluene, ethylbenzene, xylenes, mercury (Hg), nickel (Ni), chromium (Cr), lead (Pb) (environmental pollution)	*OGG1*
Sinitsky et al.	2015	Russia	Control group—Village Zarubino 14.9 ± 2.6 (8–18 years) Exposed group—Tashtagol 12.1 ± 2.32 (8–17 years)	Control group—Village Zarubino *n* = 60 (27 males and 33 females) Exposed group—Tashtagol n = 60 (34 males and 26 females)	Radon (^222^Rn) (environmental pollution)	Repair DNA genes: *ADPRT (PARP1)* *hOGG1* *NBS1* *XRCC1* *XpC* *XpD* *XpG*
Mielzynska‐Svach et al.	2013	Poland	5–14 years	*n* = 74 healthy children (47 males and 27 females)	Polycyclic aromatic hydrocarbons (occupational and environmental pollution)	*GSTT1* *GSTM1* *CYP2D6* *EPHX1* *GSTP1* *NAT2* *XPD* *XRCC1* *XRCC3*
Zeller et al.	2012	Germany	30 male smokers (26 ± 4.9 years) 30 female nonsmokers (29 ± 7.6 years) 30 school children (11 ± 0.6 years)	*n* = 90 30 male smokers 30 female nonsmokers 30 school children	Formaldehyde (150 μM)	*GSTM1* *GSTT1*
Sanchez‐Guerra et al.	2012	Mexico	6–10 years	*n* = 82 (45% boys and 55% girls)	Polycyclic aromatic hydrocarbons (environmental petrochemicals industries pollutants)	CYP1A1 CYP1B1 GSTT1 GSTM1
Sampayo‐Reyes et al.	2010	Mexico	63 children (8.1 ± 0.2 years) 61 adults (35.8 ± 1.2 years)	*n* = 124 63 children (50.8% males and 49.2% females) 61 adults (8.2% males and 91.8% females)	Environmentally arsenic contaminated region	GSTO1 AS3MT
Buthbumrung et al.	2008	Thailand	9–13 years (10.72 ± 0.11 years)	*n* = 171 109 urban boys 62 rural boys	Benzene (urban air pollution)	CYP2E1 NQO1 GSTA1 GSTM1 GSTT1

### 3.2 Variables Related to Mutagens Exposure in Children

Table 3 presents the key factors linked to environmental mutagen exposure and genotoxicity in children. Eight out of 10 studies incorporated a negative control group to guarantee precise and reliable comparisons.

**TABLE 3:** Variables assessed in the included studies in this systematic review.

Author	Cell type	Design	Mutagenic assays	*N* of evaluated units	Stain	Evaluated parameters	Inclusion criteria	Cyto‐toxicity analysis	Blind analysis	Proper statistical description	Control
Marín et al. 2024	Peripheral Blood	In vivo	Comet assay	100	Syber Green	Percentage of DNA in the tail (%Tail‐DNA)	Yes	No	No	Yes (linear regression model)	Yes
Sun et al. 2022	Urine	In vivo	RP‐HPLC (8‐OHdG and 8‐oxoG)	—	—	Levels of urinary 8‐OHdG and 8‐oxoG	Yes	No	Yes	Yes (independent *t*‐test, Shapiro–Wilk)	Yes
Chang et al. 2021	Urine	In vivo	Liquid chromatography–tandem mass spectrometry (8‐OHdG, 8‐NO2Gua, 8‐iso‐PGF2α and HNE‐MA)	—	—	Level of urinary 8‐OHdG, 8‐NO2Gua, 8‐iso‐PGF2α and HNE‐MA	Yes	No	No	Yes (chi‐square test, logistic regression model)	Yes
Montero‐Montoya et al. 2020	Peripheral blood	In vivo	Reticulocyte micronucleus test	Not informed	Thiazole orange	Flow cytometry	Yes	No	No	Yes (Kruskal Wallis)	Yes
Sinitsky et al. 2015	Peripheral blood	In vivo	Micronucleus assay (MN)	1000	Azure‐eosin	Cell counting	Yes	Yes (Index proliferation)	No	Yes (Mann–Whitney *U* test)	Yes
Mielzynska‐Svach et al. 2013	Peripheral Blood And Urine	In vivo	Micronucleus assay (MN) Sister chromatid exchange test (SCE test) Aromatic DNA adducts analysis Urinary mutagenicity (AMES)	1000 (MN) 50 (SCE test)	Giemsa (MN)	Cell counting	Yes	No	Yes (MN)	Yes (Student's *t*‐test)	Yes
Zeller et al. 2012	Peripheral Blood	Ex vivo	Alkaline Comet Assay Sister chromatid exchange test (SCE test)	100 (Comet Assay) 100 (SCE test)	Ethidium bromide (Comet Assay) Giemsa (SCE test)	Tail intensity (Comet Assay) Cell counting (SCE test)	Yes	Yes (Index proliferation)	Yes (Comet Assay)	Yes (Kruskal–Wallis test, two sample‐Wilcoxon‐tests were performed for pairwise comparisons)	Yes
Sanchez‐Guerra et al. 2012	Peripheral Blood Urine	In vivo	Comet Assay 1‐OHP	50 (comet assay)	Not informed	Olive Tail Moment	Yes	No	No	Yes (multiple linear regression analysis)	No
Sampayo‐Reyes et al. 2010	Peripheral Blood	In vivo	Comet Assay	200 (Comet Assay)	4′6‐diamidino‐2‐phenylindole	Percentage of DNA in the tail (%Tail‐DNA)	Yes	Yes (cell viability/trypan Blue)	Yes	Yes (general linear model and univariate linear model)	No
Buthbumrung et al. 2008	Peripheral blood Urine	In vivo	HPLC‐ECD (8‐OHdG)	—	—	Levels of 8‐OHdG in leukocytes and urine	Yes	No	No	Yes (general linear model)	Yes

Abbreviations: 1‐OHP = 1‐Hydroxypyrene; 8‐iso‐PGF2 = 8‐iso Prostaglandin F 2α, 8‐OHdG = 8‐Hydroxy‐2‐deoxyguanosine, 8‐NO2Gua = 8‐nitroguanine, C = control group, CBMN = cytokinesis‐blocked micronucleus, HNE‐MA = 4‐hidroxinonenal, HPLC‐ECD = high‐performance liquid chromatography with electrochemical detection, MN = micronucleus assay, N/A = not applicable, RP‐HPLC = reverse phase high‐performance liquid chromatography, SCE = sister chromatid exchange, — = not described.

* Positive control.

The presented Table 3 summarizes findings from 10 studies investigating the genotoxic effects of different mutagens on peripheral blood and/or urine samples. The methodologies for assessing genotoxicity varied across these studies. Fourutilized the Comet assay, three used the SCE assay and one utilized the micronucleus assay (MN). Besides the aforementioned tests, other assays were also used, such as AMES and HPLC‐ECD. 8‐OHdG was also assessed in some studies, as its assessment implies oxidative DNA injury. Peripheral blood emerged as the primary tissue analyzed, highlighting its significance in monitoring genotoxic effects from different mutagens exposure. Regarding the control groups, eight out of 10 studies included negative controls.

The studies assessed various indicators of DNA damage through different assays. In addition, PCR‐RFLP assays were used to identify genetic polymorphisms in genes linked to DNA repair and radiation response, including CYP1A1, GSTM1, GSTT1, XpG, XRCC1, XRCC3, PON, ADPRT, and NBS1. Cytotoxicity analyses were conducted in only three of the studies. Although most studies included statistical analyses, only four out of ten employed blinded procedures, highlighting the need for improved research design to reduce potential biases.

In terms of demographic characteristics, the studies predominantly focused on pediatric populations, with a gender distribution that included both males and females across various age ranges. Specifically, as shown in Table 1, most participants were between 3 and 13 years old, which is typical for exposure scenarios involving this pediatric group.

### 3.3 Main Results

The data presented in Table 4 consolidate key findings from multiple studies investigating the genotoxic and cytotoxic effects of mutagen exposure in children, alongside the associated genetic polymorphisms. Of the 10 studies reviewed, nine reported elevated genotoxicity, as evidenced by various biomarkers. Notably, only three studies failed to establish a significant link between specific polymorphisms and increased genotoxicity. Among the six studies that did find such associations, the identified polymorphisms predominantly occurred in genes involved in crucial biological processes such as DNA repair, detoxification, and oxidative stress response. These genes included PON, OGG1, XpG, ADPRT, NBS1, GSTM1, GSTT1, XRCC3, GSTP1, EPHX1, AS3MT, CYP2E1, and NQO1.

**TABLE 4:** Main findings of studies in chronological order of authors.

Authors	Genotoxicity assay	Cytotoxicity assay	Polymorphism	Observation
Marín et al. 2024	Children in Medellín, who live in an area with higher exposure to pollutants, had higher levels of DNA damage by comet assay when compared to those in Bogotá.	—	Genotoxic effects were not associated with polymorphisms genes	—
Sun et al. 2022	Children from high PM2,5 level campus was significantly higher than that of low PM2,5 level campus children of 8‐OHdG and 8‐oxoG urinary levels	—	Genotoxic effects were not associated with polymorphism genes	—
Chang et al. 2021	Children with ADHD had significantly higher DMP and HNE‐MA urinary level than control group children	—	Genotoxic effects were associated with polymorphism in PON gene	—
Montero‐Montoya et al. 2020	Children in Tepetitla, exposed to industrial pollution, presented higher levels of micronuclei compared to children in Ixtenco, who live in a less polluted area. Children with the GG genotype had significantly higher MN frequencies compared with the CC and CG genotypes from OGG1 gene.	—	Genotoxic effects were associated with polymorphisms in OGG1 gene.	—
Sinitsky et al. 2015	Children in Tashtagol exposed to Radon, presented higher levels of micronuclei and nucleoplasmic bridge compared to children in Village Zarubino, who live in a less radioactive area	No significant changes	Genotoxic effects were associated with polymorphisms in XpG, ADPRT, and NBS1 genes	—
Mielzynska‐Svach et al. 2013	The level of aromatic DNA adducts was approximately twice as high in children with the GSTM1 null genotype compared with those with the active gene. Children with the GSTT1 gene deletion also had higher levels of DNA adducts. Children with the EPHX1 Arg/Arg genotype had significantly higher mutagenicity by AMES test compared to those with other genotypes (His/His and His/Arg). The XRCC3 Met/Met genotype was associated with a significantly higher number of SCE in peripheral blood lymphocytes compared with Thr/Met allele carriers. Children with the slow GSTP1 genotype had a significantly higher frequency of MN in peripheral blood lymphocytes compared with those with the fast phenotype	—	Genotoxic effects were associated with polymorphisms in GSTM1, GSTT1, XRCC3 GSTP1, *EPHX1* Genes	—
Zeller et al. 2012	No significant changes	No significant changes	Genotoxic effects were not associated with polymorphisms in GSTM1 and GSTT1 genes	Ex vivo formaldehyde exposure Cells irradiated with 2 Gy gamma irradiation Formaldehyde (150 μM) caused a reduction of gamma ray‐induced DNA migration
Sanchez‐Guerra et al. 2012	High percentage of children had 1‐ to 17‐fold higher 1‐OHP concentrations than those previously reported in nonoccupationally exposed adults, representing high PAH exposure Children who lives near to petrochemical industries has more positive association between 1‐OHP and DNA damage by comet assay	—	Genotoxic effects were associated with polymorphism in CYP1A1 and GSTM1 with 1‐OHP concentrations	—
Sampayo‐Reyes et al. 2010	Positive correlation was found between urinary arsenic content and DNA damage by comet assay, individuals with higher levels of DNA damage were also those with higher level of As in urine. No significant differences in the means of values of genetic damage were observed between adults and children.	No significant changes	Genotoxic effects were associated with polymorphism in AS3MT gene, but only among children	Arsenic levels were measured in urine and drinking water
Buthbumrung et al. 2008	Level of 8‐OHdG in leukocytes was three times higher in urban children compared to rural children A significant increase in the level of urinary 8‐OHdG was also observed compared urban to rural children Leukocytes 8‐OHdG were statistically associated with benzene exposure while urinary 8‐OHdG were not statistically associated with benzene exposure	—	Genotoxic effects were associated with polymorphism genes CYP2E1 and NQO1.	

Abbreviations: ↑ = increase, ADPRT = protein(ADP‐ribosyl) transferase, AS3MT = Arsenite methyltransferase gene, CC = cytosine cytosine genotype, CG = cytosine guanine genotype, CYP, cytochrome P450, EPHX1 = Epoxide hydrolase 1, ERCC, excision repair cross complementation group 1, GSTM1 = glutathione S‐transferase mu 1, MN = micronucleus, NBS1 = nibrin gene, 1‐OHP = 1‐hydroxypirene, PM = particulate matter level, SCE = sister chromatid exchange, XpG =, XRCC1 = x‐ray repair cross complementing family.

These results emphasize the prominent role of genetic variations in key pathways like DNA repair and detoxification in shaping individual susceptibility to mutagenic exposures and, consequently, in modulating the risk of genotoxic damage. Such polymorphisms may not only affect the efficiency of DNA repair mechanisms but also influence the body's ability to detoxify harmful substances, ultimately determining the extent of genomic instability induced by environmental mutagens.

### 3.4 Quality Assessment

Table 5 presents the assessment of study quality. Of the 10 studies reviewed, six were rated as strong, three as moderate, and one as weak. This means that nine studies were classified as either Strong or Moderate. The evaluation of confounding factors considered aspects such as blind analysis, thorough statistical reporting, staining techniques, meta‐nuclear examination, cell counting, absence of dosimetry, and inclusion criteria.

**TABLE 5:** Quality assessment and final rating of the studies.

Author	Number of confounders	Details	Rating
Marín et al. 2024	2	Blind analysis and cytotoxicity	Moderate
Sun et al. 2022	1	Cytotoxicity	Strong
Chang et al. 2021	2	Blind analysis and cytotoxicity	Moderate
Montero‐Montoya et al. 2020	1	Cytotoxicity	Strong
Sinitsky et al. 2015	—	—	Strong
Mielzynska‐Svach et al. 2013	1	Cytotoxicity	Strong
Zeller et al. 2012	—	—	Strong
Sanchez‐Guerra et al. 2012	3	Blind analysis, cytotoxicity, and control	Weak
Sampayo‐Reyes et al. 2010	—	Control	Strong
Buthbumrung et al. 2008	2	Blind analysis and cytotoxicity	Moderate

## 4 Discussion

The potential for environmental mutagens to induce genetic damage in children is a significant area of concern due to the heightened vulnerability of young individuals to the harmful effects of pollutants. Children, given their developmental stage, have a greater susceptibility to environmental exposures that can alter their genetic makeup, increasing the risk of health issues such as cancer, developmental disorders, and other chronic conditions (Smith et al. 2020). Studying the interplay between environmental agents and genetic factors is vital for understanding how these exposures affect the health of children and identifying those who may be more vulnerable. This systematic review aims to evaluate whether environmental mutagens, such as air pollution, pesticides, and other industrial chemicals, can indeed cause genotoxicity in children, and to assess if certain genetic polymorphisms increase the risk of genetic damage in response to these agents.

Several of the studies reviewed shed light on the significant associations between environmental exposures and genetic damage in children. Among the most commonly studied pollutants were benzene, PAHs, arsenic, organophosphate pesticides, x‐ray, and formaldehyde. These data were expected due to the fact that it is well established that these environmental agents are able to induce genotoxicity in mammalian cells in vivo (Alarcón‐Herrera et al. 2024; Amazonas et al. 2024; Cunha et al. 2021; Costa et al. 2015). For this reason, the effects of these agents on DNA integrity, measured through biomarkers such as micronuclei, DNA adducts, oxidative stress markers, and the comet assay, demonstrate the widespread impact of environmental pollution on children's genetic material. For example, exposure to high levels of benzene in urban areas resulted in oxidative DNA damage and an increase in the levels of biomarkers indicative of genotoxicity (Buthbumrung et al. 2008). Similarly, children living near petrochemical complexes in Mexico were found to have higher DNA damage levels correlated with PAH exposure (Sanchez‐Guerra et al. 2012).

To the best of our knowledge, there are many studies investigating the role of genetic polymorphisms in children suffering many diseases, such as anemia cancer, congenital anomalies, COVID‐19 susceptibility among others (Deng et al. 2025; Muruganantham and Veerabathiran 2025; Zhang et al. 2025; Kozak et al. 2025). However, the role of genetic polymorphisms on susceptibility to environmental mutagens is still little explored in the literature. Our results demonstrated a significant aspect on the role of genetic susceptibility, particularly concerning polymorphisms in genes involved in the metabolism and repair of DNA following exposure to environmental mutagens. Many studies identified specific polymorphisms that may modulate individual responses to environmental pollutants. For instance, the GSTM1 null genotype and variations in CYP1A1, OGG1, and H2AX were found to influence the extent of DNA damage in children exposed to air pollution, PAHs, and other mutagenic compounds. These findings suggest that genetic differences in detoxification and DNA repair pathways play a crucial role in determining the extent of genotoxicity from environmental exposures in children.

The studies also highlight the importance of considering the minimum requirements for genotoxic testing. According to expert recommendations, the analysis of micronuclei should include at least 1000 cells for blood samples and all studies that used this assay followed this recommendation (Bonassi et al. 2001). Additionally, the comet assay requires a minimum of 50 cells for reliable results (Glei et al. 2016; Hartmann et al. 2003; Pogribny et al. 2018). Notably, all studies that measured micronuclei or conducted comet assays adhered to these standards, ensuring the robustness and reliability of their findings.

However, some limitations in the studies must be acknowledged. First, while the inclusion of genetic analyses provides valuable insight into individual susceptibility, the genetic polymorphisms studied were not exhaustive. The interaction between gene–environment factors is highly complex, and there may be additional unmeasured polymorphisms that could contribute to the observed genotoxic effects. Second, while most studies adhered to appropriate sample sizes and quality control measures, some studies had relatively small sample sizes, which may limit the generalizability of their findings. Moreover, many studies were cross‐sectional, making it difficult to establish causality between exposure and genetic damage.

Despite these limitations, the overall quality of the studies included in this review is high. Nine out of 10 studies were classified as strong or moderate, contributing to the overall confidence in the findings. Also, nine out of 10 reported increased genotoxicity. These studies, particularly those that utilized robust biomarkers and adhered to well‐established guidelines for genotoxic testing, offer important insights into the mechanisms through which environmental exposures contribute to genetic damage in children. The findings suggest that environmental agents such as benzene, PAHs, and arsenic pose a real risk to children's health, with genetic factors significantly influencing individual susceptibility (Li et al. 2019; Wang et al. 2020).

In conclusion, these findings underscore the complex interplay between genetic predisposition and environmental exposures, highlighting the need for further research to better understand how genetic polymorphisms in these pathways contribute to inter‐individual variability in genotoxic outcomes. Anyway, this review underscores the importance of considering both environmental exposures and genetic factors in assessing the risk of genotoxicity in children. The findings suggest the need for continued research to identify additional genetic markers of susceptibility and to better understand the long‐term health implications of environmental pollution on child development. These insights can inform public health policies aimed at reducing children's exposure to harmful pollutants and contribute to the development of genetic screening tools to identify those at greater risk of harm.

## Author Contributions

Study design: Thiago Guedes Pinto and Daniel Araki Ribeiro. Data search: Thiago Guedes Pinto, Daniel Vitor de Souza, Gabriel Carvalhal de Aguiar, and Daniel Araki Ribeiro. Data analysis: Thiago Guedes Pinto, Daniel Vitor de Souza, Gabriel Carvalhal de Aguiar, and Daniel Araki Ribeiro. Writing the paper: Thiago Guedes Pinto, Ana Claudia Muniz Renno, Patricia Ramos Cury, Daniel Vitor de Souza, Gabriel Carvalhal de Aguiar, Jean Nunes dos Santos, Rogerio Aparecido Dedivitis, and Daniel Araki Ribeiro.

## Funding

The authors acknowledge research grants received from CNPq (Conselho Nacional de Desenvolvimento Científico e Tecnológico) (Grant Number #001) for productivity fellowship.

## Conflicts of Interest

The authors declare no conflicts of interest.

## Data Availability

Data sharing is available to this article upon request.
